# The protective potential of a *Fraxinus xanthoxyloides* ethyl acetate fraction against CCl_4_-induced oxidative stress in the cardiac tissue of rats

**DOI:** 10.1039/c9ra08729j

**Published:** 2020-03-10

**Authors:** Tahira Younis, Faiza Jabeen, Laila Jafri, Azhar Rasul, Maleeha Manzoor, Mussarat Shaheen, Ammara Riaz

**Affiliations:** Department of Biochemistry, Faculty of Biological Sciences, Quaid-i-Azam University Islamabad 45320 Pakistan tahirayounis@gmail.com lailashah9@gmail.com +92 3007311813; Department of Zoology, Faculty of Life Sciences, Government College University Faisalabad 38000 Pakistan acancerian@hotmail.com drazharrasul@gmail.com maleeha.manzoor@yahoo.com shaheenmust@gmail.com ammarach21@gmail.com; Department of Microbiology, Faculty of Biological Sciences, Quaid-i-Azam University Islamabad 45320 Pakistan; Department of Life Sciences, Abasyn University Islamabad Campus Islamabad Pakistan

## Abstract

Secondary metabolites present in medicinal plants offer a golden opportunity to fight different ailments, such as cancer, infections, diabetes, neurodegenerative and cardiovascular diseases, *etc.* The traditional use of various parts of *Fraxinus xanthoxyloides* is known to serve as a cure for pneumonia, pain, jaundice, malaria, fracturing of bones, and internal wounds. The aim of this research was to validate the antioxidant and cardio-protective properties of *F. xanthoxyloides* leaves. The antioxidant potential was evaluated by employing different assays on the crude methanol extract, as well as its derived fractions. The extract/fraction that showed significant activity was further investigated for the presence of phytochemicals using high performance liquid chromatography-diode array detector (HPLC-DAD) analysis and also for cardio-protective potential. In the case of the antioxidant potential, the ethyl acetate fraction (FXE) was demonstrated to have the most potent total antioxidant (26.3 ± 2.4 AAE μg mg^−1^), hydroxyl ion scavenging (IC_50_ = 7.9 ± 0.9 μg mg^−1^), ferrous ion chelating (IC_50_ = 28.2 ± 2.7 μg mg^−1^) and nitric oxide scavenging (IC_50_ = 32.5 ± 2.9 μg mg^−1^) effects among all of the extract/fractions, whereas in the case of DPPH (IC_50_ = 17.5 ± 2.7 μg mg^−1^) and the reducing power assay (16.7 ± 2.8 GAE μg mg^−1^), promising antioxidant potential was shown by the *n*-butanol fraction. The presence of different concentrations of rutin, caffeic acid, catechin, and gallic acid was observed in the high performance liquid chromatography (HPLC) profile of FXE. Furthermore, in *in vivo* experimentation, the oral administration of FXE and silymarin significantly restored the CCl_4_-induced increase in the levels of creatine kinase, creatine kinase-MB, cholesterol and triacylglycerides when compared with the untreated group. FXE and silymarin treatment also restored the levels of the tissue antioxidant enzymes, for example glutathione-*S*-transferase, glutathione reductase, catalase, peroxidase and superoxide dismutase. Furthermore, significantly lower levels of reduced glutathione and enhanced levels of lipid peroxides, hydrogen peroxide, comet length and DNA damages were observed after CCl_4_ administration in the cardiac tissue of rats. FXE was able to restore these biochemical parameters, as well as the histological status of heart tissue. Based upon the present investigation, we concluded that *F. xanthoxyloides* leaves may have cardio-protective potential similar to silymarin against CCl_4_ induced injuries owing to its antioxidant constituents.

## Abstract 2

Secondary metabolites present in medicinal plants offer a golden opportunity to fight different ailments, such as cancer, infections, diabetes, neurodegenerative and cardiovascular diseases, *etc.*

## Graphical abstract

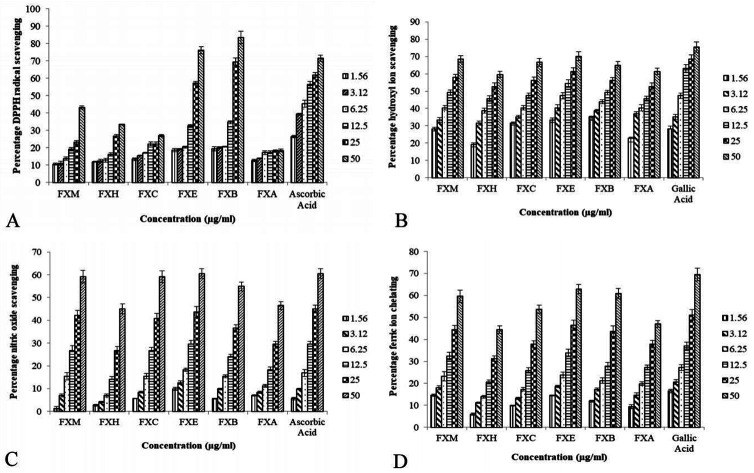

## Introduction

Plant-based natural products continue to provide key scaffolds for the development of drugs against various diseases.^1^ During the last decade, utilization of traditional plant-based medicines has been expanded globally.^2^ According to a report by the World Health Organization, 80% of the population of the world still depend upon herbal medicine.^1^

Reactive oxygen species (ROS) such as O_2_˙^−^, H_2_O_2_, OH and peroxynitrate play a vital role in the cardiovascular system. They are constantly generated by the cells as an integral constituent of cellular homeostasis and are stabilized by extra- and intracellular antioxidants. If the production and counter stabilization (by antioxidants) of ROS loses its balanced state, the cardiovascular system suffers oxidative stress. ROS have significant potential to induce oxidative damage or modify the DNA, cellular proteins and lipids with detrimental consequences for the vascular structure and function.^3,4^ Chemical tissue toxicity induced by CCl_4_ is a conventional model for ROS generation in the bodily organs such as the lungs, heart, brain, testes and kidneys. Followed by the intake of CCl_4_, it is readily transformed into the trichloromethyl (CCl_3_) free radical by cytochrome P_450_ in the liver, thus causing lipid peroxidation, DNA damage or protein injuries. The trichloromethyl radical has the potential to interact with oxygen in its molecular form to give peroxy trichloromethyl radicals producing even more deleterious lipid peroxy radicals and causing massive damage to DNA and proteins. Thus, CCl_4_ provokes the production of lipoperoxides and unsaturated fatty acids which is an ultimate consequence of various pathologies.^5^ The curing of oxidative stress-induced pathological conditions with naturally occurring antioxidants is an important issue these days. Herbal antioxidants have the striking potential to protect against cardiovascular damage by strengthening the antioxidant defense systems.^6^ The evident risk of neurodegenerative and cardiovascular ailments can be highly limited by a dietary intake of fruits, vegetables and tea which act as natural antioxidants.^7^

Silymarin, a plant-derived active entity has been isolated from *Silybum marianum*, a medicinal plant. It is a mixture of three natural flavonoids, these are silybin, silydianin and silychristin. The leaves, fruits and seeds are the reservoirs of silymarin in this plant.^8^ Silymarin has shown a protective potential to cure liver disorders^9^ and myocardial infarction caused by reperfusion^10^ in rats. It can also scavenge oxygen free radicals and prevent lipid peroxidation.^11^ The activity of silymarin against inflammation can be attributed to its potential to inhibit the action of NF-κB which is required for the induction of a large number of pro-inflammatory mediators such as IL-1, IL-6 and TNF-α.^12^ Silymarin has shown protective effects against chemical induced cardiotoxicity.^13^

*Fraxinus xanthoxyloides* (G. Don) Wall. ex A.DC.^14^ (family Oleaceae), a traditional medicinal plant, is commonly found in Pakistan, India, Algeria and Morocco. In Pakistan, the local practitioners utilize different parts of this plant to cure patients of malaria, pneumonia and jaundice. Decoction from the bark of its stem is utilized by local people for the reduction of labor pain^15^ and bone fractures.^16^ Being enriched with a variety of phytochemicals such as phenylethanoids, flavonoids, secoiridoids, lignins, and coumarins, the genus *Fraxinus* is reported in literature as an anticancer, renoprotective, antioxidant, anti-inflammatory, and anti-diabetic agent.^17^

This experimental research was performed to prove the potential efficacy of the leaves of *Fraxinus xanthoxyloides* against CCl_4_-induced cardiotoxicity in rats and, as far as the authors are aware, the cardio-protective potential of this plant has not yet been determined. To address this objective, we investigated the antioxidant potential of the *F. xanthoxyloides* leaves. Following this step, high performance liquid chromatography-diode array detector (HPLC-DAD) analysis was performed to probe the type of phytochemicals, especially polyphenols and flavonoids, present in this plant. *In vivo* studies were performed and the cardiac tissue was investigated for serum biomarkers, antioxidant activity, DNA damage and histopathology.

## Materials and methods

### Plant material

*F. xanthoxyloides* leaves were collected from Margalla Hills (Islamabad) Pakistan, in November 2016. We referred to the flora of Pakistan and sought the guidance of Dr M. Zafar in the Department of Plant Sciences, Quaid-i-Azam University (QAU) Islamabad to identify the plant. A token specimen of the plant (45680) was placed at National Herbarium, QAU, Islamabad, Pakistan.

### Preparation of crude extract and fractions

The leaves were air-dried under shade after rinsing with water, for three weeks. With the help of an electric grinder the leaves were ground and then by mixing 3 kg of the powdered leaves with 9 L of 95% crude methanol, an extraction was carried out. The powder was kept submerged at room temperature for a time period of one week, followed by filtration (using Whatman #1 filter paper). The extraction process was repeated twice. The filtrates obtained were mixed and processed at 40 °C under low pressure on a rotary evaporator apparatus (Panchun Scientific Co., Kaohsiung, Taiwan) yielding a final mass of 287 g crude methanol extract (FXM). The next process was to resolve the compounds on the basis of polarity in an ascending manner. For this, the FXM (287 g) was suspended in 600 ml of dH_2_O, following the suspension, a liquid–liquid partition was performed by the addition of *n*-hexane (600 ml). After 1 h, the *n*-hexane layer became separated from the aqueous layer, this was then separated and concentrated using a rotatory evaporator. The same procedure was repeated with other solvents such as chloroform, ethyl acetate, and *n*-butanol. The fractionation procedure was performed three times with each solvent. Finally, we obtained 55.32 g of the *n*-hexane fraction (FXH), 36.00 g of chloroform (FXC), 27 g of ethyl acetate (FXE) and 38.46 g of the *n*-butanol (FXB) fraction. Lastly, 129.96 g of the aqueous residual layer (FXA) was obtained and these extract/fractions were then stored in a refrigerator at 4 °C for further investigation.

### *In vitro* antioxidant studies

Antioxidant assays were performed *in vitro* using different extract/fractions ranging from 0–50 μg ml^−1^. To evaluate the extent of the DPPH radical scavenging, the hydroxyl ion (OH) scavenging activity, the ferrous ion (Fe^2+^) chelating power and the nitric oxide (NO) scavenging potential of the *F. xanthoxyloides* extract/fractions, the protocols previously published by Brand-Williams *et al.*,^18^ Ilavarasan *et al.*^19^ and Dastmalchi *et al.*^20^ were followed respectively. Ascorbic acid and gallic acid were used as the standards for this purpose, respectively. The antioxidant potential was evaluated using the following equation:
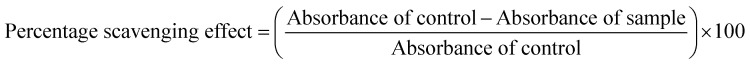


To assess the total antioxidant capability (TAC) we followed the protocol presented by Umamaheswari and Chatterjee.^21^ The TAC of each sample was measured as mg of the ascorbic acid equivalent/mg of sample (AAE μg per mg). The protocol devised by Fejes *et al.*^22^ was consulted for the estimation of the reducing power of the plant extract/fractions to convert the Fe^3+^ ions to Fe^2+^ ions. The reducing capacity of each sample was recorded as the mg of gallic acid equivalent/mg of extract (GAE μg per mg).

### High performance liquid chromatography analysis

The HPLC analysis of FXE was performed using HPLC-DAD (Agilent 1200, Germany) equipped with a Zorbax RXC8 (Agilent, USA) analytical column with a 5 μm particle size and a 25 ml capacity using the previously reported method by Zu *et al.*^23^ Each sample was diluted with HPLC grade methanol. The mobile phase consisted eluent A, (acetonitrile–methanol–water–acetic acid/5 : 10 : 85 : 1)-and eluent B (acetonitrile–methanol–acetic acid/40 : 60 : 1). The gradient (A : B) utilized was as follows: 0–20 min (0 to 50% B), 21–25 min (50 to 100% B), 26–30 min (100% B) and 31–40 (100 to 0% B) at a flow rate of 1 ml min^−1^. The standards and samples were prepared in HPLC grade methanol (1 mg ml^−1^), filtered through a 0.45 μm-membrane filter and 20 μl was injected for the analysis. Myricetin, quercetin, and kaempferol were investigated at the wavelength of 368 nm, gallic acid and rutin acid were analyzed at 257 nm, catechin was examined at 279 nm and caffeic acid was evaluated at 325 nm. Before each column was run the column was reconditioned for 10 min and the analysis was performed in triplicate for each one of the FXE samples. By using the external standard method, we have fully assimilated the peaks for the quantification of samples. All work was carried out at ambient temperature.

### Animal studies

#### Acute toxicity studies

Acute toxicity tests were performed under the 425 guidelines promoted by the Organization for Economic Cooperation and Development (OECD, 2001). A 50 mg kg^−1^ dose of DMSO was administered to three male *Rattus norvegicus* (Sprague-Dawley rats) intragastrically for 14 d, no mortality was observed and thus DMSO was used as the solvent to dissolve the extract/fractions sample. Male Sprague Dawley rats (SD rats) (*n* = 3) were subjected to various doses of the FXE, such as 50, 250, 500, 1000, and 2000 mg kg^−1^ par oral, while the control group was administered saline (10 ml kg^−1^ body weight (bw)). These experimental rats were observed daily for 14 d and their behavioral patterns, such as their salivation, sleep, lethargy, altered physical appearance, pain, and pathological signs (purgation, diarrhea, nasal secretions, lesions, lachrymation, and piloerection) as well as the mortality were recorded. No mortality appeared at 2000 mg kg^−1^ (the highest dose), instead the doses, 150 mg kg^−1^ and 300 mg kg^−1^ bw were evaluated to study the activities related to the cardio-protective potential.^24^

### Experimental design

The defensive impacts of FXE on the CCl_4_ induced cardiotoxicity were studied in male *Rattus norvegicus* (Sprague-Dawley rats) rats having a body weight range of 150–200 g. 48 experimental rats were divided into eight groups and each group comprised six rats. They were kept at the Primate Facility of QAU, Islamabad in cages with a 12 hour light/dark cycle. The study was performed by strictly following the National Institute of Health (NIH), Islamabad guidelines and approval of the experiment protocol (Bch#281) was granted by the Ethical Committee of Quaid-i-Azam University, Islamabad. The experimental rats were given rodent chow and tap water *ad libitum* as feed. The protocol devised by Shyu *et al.*^25^ was followed with a few modifications. The distribution of rats was according to the following scheme: Group I; control (untreated), Group II; vehicle control (1 ml kg^−1^ bw; 10% DMSO in olive oil, oral), Group III; CCl_4_-treated (1 ml kg^−1^ bw; 30% CCl_4_ in olive oil) intraperitoneally (30 days or 15 dosages) on alternative days, Group IV; silymarin-treated as a reference chemical (100 mg kg^−1^ bw, oral) and Group V and VI; FXE (150, 300 mg kg^−1^ bw, oral) on alternative days with CCl_4_ treatment (15 dosages), Group VII and VIII: FXE alone (150, 300 mg kg^−1^ bw) was administered orally for 30 days or 15 dosages. When the experimental period of 30 days was completed, normal feed without any treatment was given to the rats for 24 h. After that, ether was used for anesthesia and the animals were euthanized and blood was collected from the heart. Centrifugation (500 × *g* for 15 min) was performed on these blood samples while maintaining the temperature at 4 °C. After this step, serum was collected to conduct different biochemical investigations. The heart tissue was homogenized, washed with ice cold saline and the detritus was removed. One portion of the heart tissue was stored in liquid nitrogen, to conduct biochemical investigations and for DNA damage assessment. For histopathology a second portion of the heart tissue was stored in 10% phosphate buffered formalin.

### Serum analysis

To estimate the levels of serum biomarkers, creatine kinase (CK) and creatine kinase-MB (CK-MB), standard AMP diagnostic kits were utilized. The serum cholesterol and triglyceride concentration was estimated by using a reagent kit purchased from Reactivos Spinreact Company (Spain).

### *In vivo* antioxidant assays

The heart tissue from each animal was homogenized in 100 mM potassium phosphate buffer (1.5 ml) in 1 mM ethylene diamine tetra acetate (EDTA) with the pH adjusted to 7.4. Samples were homogenized and centrifuged at 12 000 × *g* at 4 °C for a period of 30 min and the supernatant was obtained, which was later used to perform antioxidant enzyme assays.

For estimation of the catalase (CAT) activity, we followed the method previously published by Chance and Maehly.^26^ An absorbance change of 0.01 units per min was defined as the catalase activity for 1 unit. The same method was followed to assess the peroxidase activity (POD) but some modifications were also made. After 1 min, the change in the OD_470_ was measured. One unit of POD activity was equivalent to 0.01 units per min. To conduct superoxide dismutase activity (SOD) the method published by Kakkar *et al.*^27^ was followed. The amount of chromogen formed as the result of the reaction was assessed by determining the intensity of color at a wavelength of 560 nm and this finding was accounted for in terms of the units per mg protein. Then, the method previously reported by Habig *et al.*^28^ was employed to evaluate the activity of the glutathione-*S*-transferase activity (GST). An altered absorbance was observed at 340 nm. The enzyme activity was evaluated as nM CDNB conjugate made/min per mg protein and the molar extinction coefficient used here was 9.6 × 10^3^ M^−1^ cm^−1^. Furthermore, the procedure by Khan *et al.*^29^ was followed for the estimation of the glutathione reductase (GSR) levels. The activity of GSR depends upon the conversion of oxidized glutathione to reduce glutathione by the consumption of NADPH. The absorbance was measured for 20 min at a wavelength of 340 nm and at a temperature of 25 °C. At a 6.22 × 10^3^ M^−1^ cm^−1^ coefficient of molar excitation, the activity by GSR was determined as the NADPH oxidized/min per mg protein.

### Biochemical parameters

The reduction in glutathione estimation (GSH) was calculated by following the protocol previously reported by Jollow *et al.*^30^ The color change was observed at the absorbance value of 412 nm. The enzyme action was measured as μM GSH per g tissue. Thiobarbituric acid reactive substances (TBARS) estimation was performed for each sample using the protocol reported by Iqbal *et al.*^31^ The TBARS formed in each of the samples were measured on a spectrophotometer while measuring the absorbance at 535 nm against a blank and were expressed as nM TBARS per min per mg tissue at 37 °C using the coefficient of molar extinction of 1.56 × 10^5^ M^−1^ cm^−1^. Soluble proteins of the tissues were assessed using the process previously published by Lowry *et al.*^32^ Heart tissue (80 mg) was homogenized in a phosphate buffer with a pH value of 8.0 and then centrifuged at 10 000 rpm (20 min) while maintaining the temperature at 4 °C. After this the supernatant (100 μl) was taken and mixed with an alkaline copper solution (1 ml). The mixture was incubated for 10 min and after that a Folin–Ciocalteu phenol reagent (100 μl) was added and the reaction mixture was shaken vigorously and then incubated for 30 min. At 630 nm the absorbance was evaluated and the total soluble protein in the cardiac tissue was measured using a standard curve of bovine serum albumin. To evaluate the quantity of H_2_O_2_, the protocol introduced by Pick and Keisari^33^ was followed. The optical density was taken against a blank at 610 nm. The quantity of liberated H_2_O_2_ was recorded as nM H_2_O_2_ per min per mg tissue by taking the H_2_O_2_ oxidized phenol red standard curve.

### DNA injuries

#### Comet assay

To assess the DNA injuries, a comet assay protocol devised by Dhawan *et al.*^34^ was used. Autoclaved slides were infused in 1% normal melting point agarose and were solidified. A tiny section of the renal tissue was placed in a chilled 1 ml lysis solution then homogenized and mixed with low melting point agarose (75 μl). The resulting mixture was then coated onto the pre-coated slides, then cover slips were placed over them. The gel was then solidified by placing the slide on ice-cubes for approximately 8–10 min. Then, the cover slip of the slide was gently detached and agarose was added one more time and allowed to solidify by placing the slides on ice cubes. A slide with three coatings of low melting agarose gel was dipped in the lysis solution for almost 10 min and then placed in the refrigerator for about 2 h. Then, electrophoresis was performed and the slides were stained with 1% ethidium bromide, after that they were visualized under a fluorescent microscope. To assess the intensity of the DNA damage, CASP 1.2.3.b image analysis software was used and 70–100 cells were observed for various comet parameters in the head of the cardiac cell nuclei.

#### DNA fragmentation assay

The DNA damage in the cardiac tissue was observed using the protocol devised by Wu *et al.*^35^ To the cardiac tissue homogenate (100 μl), Tris Triton EDTA (500 μl) was added, centrifuged (10 min) at 200 × *g* and 48 °C and the tube was labelled (B). The supernatant obtained was transferred to another tube labelled as (S) and again centrifuged (10 min) at 20 000 × *g* and 48 °C. The intact chromatin obtained was collected in tube (C). 1.0 ml of 25% TCA was added to all of the tubes (B, S and C) and incubated at 48 °C for 12 h. Following overnight incubation, the mixture was suspended at 18 000 × *g* and 48 °C and the precipitated DNA was collected. Next 5% TCA (160 μl) was added to each tube, this was then heated (20 min) and then diphenylamine solution (320 μl) was added. The tubes were vortexed and then incubation was performed for 4 h at 37 °C. The absorbance was measured at 600 nm spectrophotometrically. The percentage of fragmented DNA was measured using the formula:
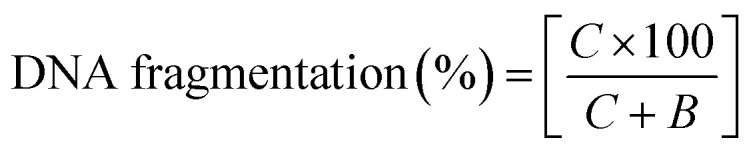


### Cardiac histopathology

For cardiac histopathological examination, the heart tissues were paraffin embedded after fixation in a fixative solution consisting of 10% phosphate buffered formalin. Thin sections (4–5 μm) of fresh cardiac tissue were prepared and staining was performed with hematoxylin/eosin. Slides were visualized under a microscope (Nikon Eclipse E100LED MVR, Japan) at 40× magnification.

### Statistical analysis

Data values are mentioned in terms of the mean ± SD. The IC_50_ was calculated by using Graph Pad Prism 4.0 in the case of the *in vitro* antioxidant activities. For the *in vivo* studies Statistix 8.1 was used for multiple comparisons among treatments using Tukeys' honestly significant difference (HSD) test and the superscript letters show the significance at *p* < 0.01. A Dunnet test with significant values at *p < 0.05, ^#^*p* < 0.01 and ^†^*p* < 0.001 was used to compare the different treatment groups with the control.

## Results

### *In vitro* antioxidant activities of FXM and its fractions

**Fig. 1:**
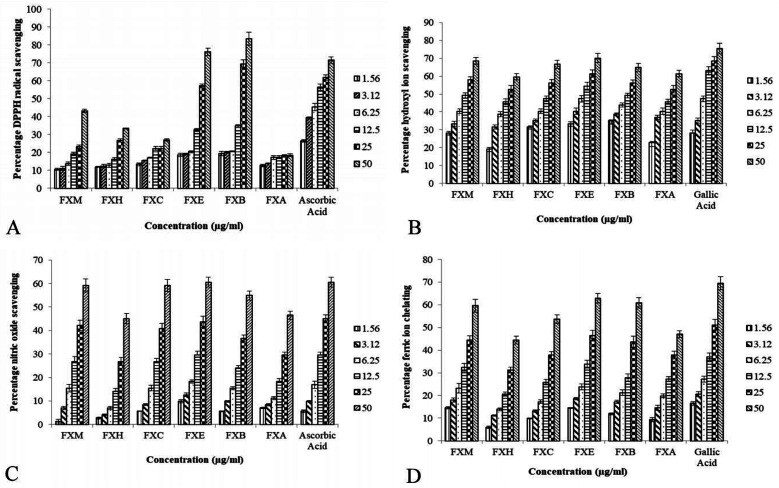
The impact of various FXM concentrations and the different fractions as indicated by several radical scavenging assays.

Table 1 and Fig. 1 depict the IC_50_ values and percentage inhibition respectively for different *in vitro* antioxidant activities. In our experiment the foraging effect of the DPPH radical varied considerably in various extract/fractions. A rise in the DPPH antioxidant activity was observed with the upsurge in concentration. The scavenging activity of the extract/fractions on the DPPH radical was noted to follow the order FXB > FXE > FXM > FXH > FXC > FXA. The most significant activity was shown by FXB (IC_50_ = 17.5 ± 2.7 μg ml^−1^) as compared to ascorbic acid IC_50_ = 8.8 ± 1.3 μg ml^−1^. Among all of the *F. xanthoxyloides* crude extract/fractions FXE showed the lowest IC_50_ = 7.9 ± 0.9 μg ml^−1^ for hydroxyl ion scavenging by revealing the best activity, followed by FXB > FXM > FXC > FXA > FXH. Compared to these, gallic acid showed an IC_50_ of 7.0 ± 1.2 μg ml^−1^ at the same concentration. The highest ferrous ion chelation was shown by FXE (IC_50_ = 28.2 ± 2.7 μg ml^−1^) compared to gallic acid (IC_50_ = 21.2 ± 2.9 μg ml^−1^) which was used as the standard. In the rest of the tested samples the scavenging activity was found to decrease in the following order, FXM > FXB > FXC > FXA > FXH. According to our results, the best nitric oxide scavenging activity was manifested by FXE (IC_50_ = 32.5 ± 2.9 μg ml^−1^) which was approximately equal to ascorbic acid (IC_50_ = 31.9 ± 3.5 μg ml^−1^) followed by FXM > FXC > FXB > FXH > FXA. An increased percentage scavenging was noticed with the increase in concentration from 0–50 μg ml^−1^.

**Table 1:** IC_50_ values of various antioxidant activities of FXM and its fractions^a^

Sample	DPPH assay	IC_50_ (μg ml^−1^)			TAC expressed as AAE μg per mg	Ferric ion reducing power expressed as GAE μg per mg
		Hydroxyl ion scavenging	Ferrous ion chelation	Nitric oxide scavenging		
FXM	47.3 ± 4.8^c^	12.4 ± 2.1^bc^	32.6 ± 3.4^d^	34.5 ± 3.5^c^	22.6 ± 2.1^b^	14.6 ± 2.4^b^
FXH	79.1 ± 5.2^b^	19.5 ± 1.3^a^	71.5 ± 5.1^a^	60.2 ± 3.3^a^	6.5 ± 2.9^d^	5.5 ± 0.7^c^
FXC	>100^a^	13.0 ± 2.0^b^	45.5 ± 3.0^c^	35.2 ± 2.8^c^	11.7 ± 5.0^c^	5.2 ± 1.1^c^
FXE	20.2 ± 3.1^d^	7.9 ± 0.9^d^	28.2 ± 2.7^e^	32.5 ± 2.9^c^	26.3 ± 2.4^a^	16.2 ± 3.1^a^
FXB	17.5 ± 2.7^e^	11.3 ± 1.1^c^	33.1 ± 3.5^d^	42.7 ± 2.1^b^	12.5 ± 1.3^c^	16.7 ± 2.8^a^
FXA	>100^a^	17.3 ± 2.5^a^	59.42 ± 3.4^b^	63.3 ± 3.9^a^	8.5 ± 1.6^d^	6.2 ± 2.0^c^
Ascorbic acid	8.8 ± 1.3^d^			31.9^3^ ± 3.5^c^		
Gallic acid		7.03 ± 1.2^d^	21.2 ± 2.9^f^			

a Data values are shown as mean ± SD (*n* = 3). Different superscripts (a–f) in each column indicate difference at the *p* < 0.01 level.

The TAC was articulated as the number of ascorbic acid equivalents (AAE) (μg per mg of extract). In our investigation TAC was seen to decline in the following order, FXE > FXM > FXB > FXC > FXA > FXH. Our results showed that FXB and FXE have the highest reducing capability with 16.7 ± 2.8 and 16.2 ± 3.1 μg GAE per mg sample respectively (assessed at 0–50 μg ml^−1^) of the extract concentration followed by FXM > FXA > FXH > FXC.

### HPLC-DAD analysis of FXE

**Fig. 2:**
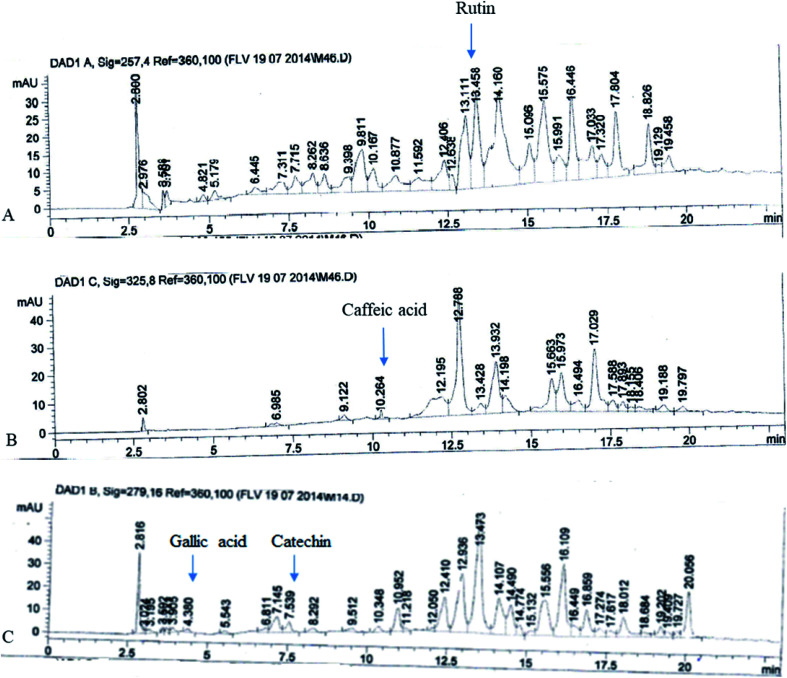
HPLC chromatograms of FXE: (A) rutin; (B) caffeic acid; and (C) gallic acid and catechin.

The FXE showed the most potent *in vitro* antioxidant activity therefore we performed the qualitative analysis of the FXE fraction of *F. xanthoxyloides* by employing reverse phase HPLC and their chromatographic sketch was related to the retention times as well as the absorption spectra of the standards to be referred (quercetin, gallic acid, caffeic acid, kaempferol, catechins, rutin, and myricetin). It was noted from the HPLC profile that FXE possesses 54.23 ± 3.41 μg/10 mg of rutin, 7.34 ± 0.93 μg/10 mg of caffeic acid, 8.45 ± 1.12 μg/10 mg of gallic acid and 12.67 ± 2.24 μg/10 mg of catechin (Fig. 2).

### Protective effects of FXE on cardiac toxicity

#### Effect of FXE on serum parameters

Table 2 displays the effect of FXE on the serum parameters, CCl_4_ treatment (*p* < 0.001) enhanced the quantity of CK and CK-MB remarkably. A remarkable (*p* < 0.001) rise in blood cholesterol and triacylglycerol was also observed after CCl_4_ treatment compared with that of the control group. Multiple comparisons amongst the treatments showed that these CCl_4_ induced elevated levels were reduced significantly in the FXE and silymarin treated groups. Although FXE alone did not show any change in the levels of the above mentioned parameters.

**Table 2:** Impact of FXE on the biochemical parameters of serum in rats^a^

Treatment	Cholesterol (mg dl^−1^)	Triacylglycerides (mg dl^−1^)	CK (U l^−1^)	CK-MB (U l^−1^)
Control	52.96 ± 1.65^d^	43.60 ± 2.10^cd^	82.00 ± 4.50^c^	93.33 ± 4.63^c^
DMSO + olive oil	52.40 ± 1.60^d^	42.03 ± 2.71^d^	87.00 ± 2.64^c^	98.00 ± 5.00^c^
CCl_4_ + olive oil	113.73 ± 3.91^†a^	84.50 ± 2.33^†a^	249 ± 37.17^†a^	299.67 ± 18.17^†a^
CCl_4_ + silymarin	79.30 ± 1.25^†b^	63.40 ± 2.49^†b^	96.00 ± 6.00^bc^	117.67 ± 6.11^bc^
CCl_4_ + FXE (150)	84.93 ± 3.38^†b^	68.43 ± 2.23^†b^	143.33 ± 11.06^†b^	160.33 ± 14.57^†b^
CCl_4_ + FXE (300)	63.50 ± 1.80^†c^	50.60 ± 2.82^#c^	86.67 ± 10.69^c^	98.00 ± 7.00^c^
FXE (150)	52.53 ± 1.10^d^	43.30 ± 1.57^cd^	85.67 ± 1.52^c^	87.33 ± 6.50^c^
FXE (300)	54.03 ± 2.47^d^	42.73 ± 1.05^d^	82.00 ± 3.00^c^	90.67 ± 6.02^c^

a Data values expressed as mean ± SD (*n* = 6). Different superscripts (a–d) in each column indicate difference at the *p* < 0.01 level. For the Dunnet comparison of treatments with the control one-way analysis of variance (ANOVA) was followed at: *p < 0.05, ^#^*p* < 0.01 and ^†^*p* < 0.001.

#### Effect of FXE on cardiac antioxidant enzymes

We observed that the administration of CCl_4_ to rats led to a substantial (*p* < 0.001) reduction in the antiradical enzyme activity, such as POD, CAT, GST, GSR and SOD when matched to the control group (Table 3). Preventive exposure of FXE and silymarin causes a reduction in the lethal effects of CCl_4_ and the activity of the above mentioned enzymes was restored towards that of the control group. We also observed that the low dosage of FXE (150 mg kg^−1^) was less potent as compared to the control group. A high dose of FXE (300 mg kg^−1^) and silymarin restored the potential efficacy of all antioxidant enzymes (except SOD) significantly. However, the use of FXE alone at the doses 150 and 300 mg kg^−1^ did not induce significant changes in the level of antioxidant enzyme activity when matched to the control.

**Table 3:** Impact of FXE on the antioxidant enzymes of cardiac tissues in rats^a^

Treatment	CAT (U l^−1^)	POD (U l^−1^)	SOD (mg dl^−1^)	GST (mg dl^−1^)	GSR (mg dl^−1^)
Control	5.54 ± 0.06^a^	11.46 ± 0.12^a^	4.56 ± 0.06^a^	160.04 ± 5.14^a^	205.39 ± 5.03^a^
DMSO + olive oil	5.66 ± 0.04^a^	11.29 ± 0.36^a^	4.64 ± 0.04^ab^	162.85 ± 5.17^a^	200.55 ± 5.02^a^
CCl_4_ + olive oil	1.3 ± 0.03^†c^	6.44 ± 0.04^†d^	1.36 ± 0.03^†e^	90.23 ± 3.54^†c^	123.75 ± 3.15^†c^
CCl_4_ + silymarin (100)	5.49 ± 0.08^a^	11.05 ± 0.04^a^	3.66 ± 0.06^bcd^	165.36 ± 2.44^a^	183.69 ± 4.00^†b^
CCl_4_ + FXE (150)	3.39 ± 0.06^†b^	8.21 ± 0.08^†c^	4.42 ± 0.09^†cd^	148.01 ± 3.04^†b^	185.45 ± 4.41^†b^
CCl_4_ + FXE (300)	5.55 ± 0.16^a^	11.09 ± 0.10^a^	4.41 ± 0.05^†d^	159.85 ± 4.64^a^	199.72 ± 4.26^a^
FXE (150)	5.46 ± 0.43^a^	11.17 ± 0.56^a^	4.53 ± 0.03^abc^	164.02 ± 4.60^a^	200.27 ± 5.87^a^
FXE (300)	5.47 ± 0.05^a^	11.28 ± 0.04^b^	4.52 ± 0.05^bcd^	161.64 ± 2.83^a^	201.61 ± 3.21^a^

a Data values expressed as mean ± SD (*n* = 6). Different superscripts (a–d) in each column indicate difference at the *p* < 0.01 level. For the Dunnet comparison of treatments with the control one-way ANOVA was followed at: *p < 0.05, ^#^*p* < 0.01 and ^†^*p* < 0.001.

#### Effect of FXE on biochemical markers of the heart

The consequences of FXE on the biochemical factors of the heart against CCl_4_ are described in Table 4. CCl_4_ treatment amplified the levels of H_2_O_2_, TBARS and DNA injuries and reduced (*p* < 0.001) the content of the proteins and GSH in the heart tissues, as with the control group. Administration of FXE reduced the concentration of TBARS, H_2_O_2_ and DNA injuries in comparison to the CCl_4_ group. Multiple comparisons of different treatments were performed to assess the restoring capability of FXE (300 mg kg^−1^) and silymarin. The restoration potential of FXE and silymarin on the protein, TBARS and H_2_O_2_ contents towards the control were significantly similar to one another, but in the case of DNA and GSH, silymarin failed to restore the protective potential to that of FXE. Administration of FXE (150 and 300 mg kg^−1^) alone did not cause modifications (*p* > 0.05) in the levels of the aforementioned biochemical markers of the heart as matched to the normal group.

**Table 4:** Impact of FXE on the biochemical parameters of cardiac tissues in rats^a^

Treatment	Protein	GSH (U l^−1^)	TBARS (U l^−1^)	H_2_O_2_ (mg dl^−1^)	DNA
Control	3.02 ± 0.03^a^	20.59 ± 1.22^ab^	3.23 ± 0.05^b^	1.19 ± 0.06^d^	19.72 ± 0.16^cde^
DMSO + olive oil	2.96 ± 0.09^bc^	21.33 ± 0.46^a^	3.34 ± 0.03^b^	1.16 ± 0.03^d^	19.29 ± 0.47^e^
CCl_4_ + olive oil	1.02 ± 0.02^†e^	10.09 ± 0.06^†d^	6.69 ± 0.06^†a^	2.6 ± 0.06^†a^	49.26 ± 0.69^†a^
CCl_4_ + silymarin (100)	3.03 ± 0.03^a^	16.47 ± 0.38^†c^	3.17 ± 0.07^b^	1.16 ± 0.04^d^	24.72 ± 0.05^†b^
CCl_4_ + FXE (150)	2.81 ± 0.05^†c^	19.4 ± 0.64^#b^	3.30 ± 0.31^b^	1.7 ± 0.03^†b^	20.26 ± 0.31^c^
CCl_4_ + FXE (300)	3.05 ± 0.03^b^	20.35 ± 0.20^ab^	3.28 ± 0.04^b^	1.32 ± 0.02^†c^	20.23 ± 0.18^cd^
FXE (150)	3 ± 0.03^bc^	21.23 ± 0.37^a^	3.11 ± 0.06^b^	1.22 ± 0.02^d^	19.45 ± 0.32^de^
FXE (300)	3.01 ± 0.19^a^	20.81 ± 0.55^a^	3.11 ± 0.40^b^	1.18 ± 0.03^d^	19.89 ± 0.16^cde^

a Data values expressed as mean ± SD (*n* = 6). Different superscripts (a–e) in each column indicate difference at the *p* < 0.01 level. For the Dunnet comparison of treatments with the control one-way ANOVA was followed at: *p < 0.05, ^#^*p* < 0.01 and ^†^*p* < 0.001.

#### Effect of FXE on comet parameters

**Fig. 3:**
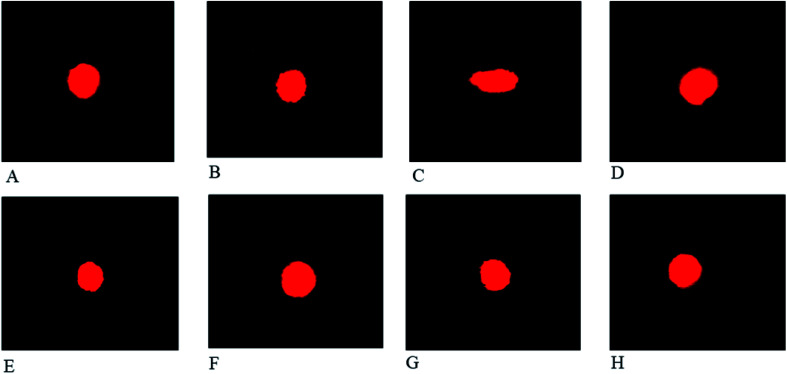
Comet analysis of the heart tissues in the presence of ethidium bromide stain (40×): (A) control; (B) vehicle treated (DMSO + olive oil); (C) CCl_4_ + olive oil treated; (D) CCl_4_ + silymarin (100 mg kg^−1^) treated; (E) CCl_4_ + FXE (150 mg kg^−1^) treated; (F) CCl_4_ + FXE (300 mg kg^−1^) treated; (G) FXE (150 mg kg^−1^) treated; and (H) FXE (300 mg kg^−1^) treated rats.

We observed that CCl_4_ treatment potentiated DNA damage in heart cells and considerably (*p* < 0.001) amplified the comet length, head length, tail length, DNA percentage in the tail and the tail moment compared to that of the control group (Table 5). It was noted that the percentage of DNA in the head declined ominously (*p* < 0.001) as compared to the control group. FXE usage relieved the lethal actions induced by CCl_4_ and modulated the values of the aforementioned factors to that of the untreated group. Multiple comparisons of different treatments indicated that a greater dosage of FXE (300 mg kg^−1^) considerably (*p* < 0.01) reestablished the comet parameters. Administration of FXE alone did not modify (*p* > 0.05) the extent of the comet variables when matched with the control group. The protective effects of FXE on the comet parameters after CCl_4_ induced toxicity in the heart tissue of experimental rats are also presented in Fig. 3 after ethidium bromide staining.

**Table 5:** Impact of FXE on the comet parameters of heart cells in rats^a^

Treatment	Comet length	Head length	Tail length	DNA head	DNA tail	Tail moment
Control	29.28 ± 1.07^b^	28.23 ± 0.77^bc^	1.06 ± 1.36^b^	97.73 ± 0.99^a^	2.26 ± 0.99^d^	0.52 ± 0.02^c^
DMSO + olive oil	29.99 ± 0.72^b^	27.27 ± 0.86^c^	2.71 ± 1.00*^b^	97.06 ± 0.95^a^	2.94 ± 0.95^d^	0.53 ± 0.00^c^
CCl_4_ + olive oil	61.88 ± 1.16^†a^	41.51 ± 1.91^†a^	20.36 ± 1.76^†a^	68.67 ± 1.14^†d^	31.33 ± 1.14^†a^	1.87 ± 0.06^†a^
CCl_4_ + silymarin (100)	30.35 ± 0.56^b^	29.18 ± 0.38^bc^	1.17 ± 0.55^b^	91.18 ± 1.11^†b^	8.82 ± 1.11^†c^	0.54 ± 0.02^b^
CCl_4_ + FXE (150)	30.58 ± 0.78^b^	29.94 ± 0.88*^b^	0.63 ± 0.62^b^	85.07 ± 0.84^†c^	14.93 ± 0.84^†b^	0.54 ± 0.02^b^
CCl_4_ + FXE (300)	29.81 ± 0.76^b^	28 ± 1.09^bc^	1.81 ± 0.78^b^	96.32 ± 0.60^a^	3.67 ± 0.60^d^	0.53 ± 0.01^b^
FXE (150)	30.10 ± 0.67b	28.72 ± 0.74bc	1.38 ± 0.83b	96.91 ± 0.76a	3.08 ± 0.76d	0.54 ± 0.01b
FXE (300)	29.85 ± 0.73b	28.85 ± 0.60bc	1.00 ± 0.45b	96.20 ± 0.56*a	3.79 ± 0.56*d	0.53 ± 0.01b

a Data values expressed as mean ± SD (*n* = 6). Different superscripts (a–d) in each column indicate difference at the *p* < 0.01 level. For the Dunnet comparison of treatments with the control one-way ANOVA was followed at: *p < 0.05, ^#^*p* < 0.01 and ^†^*p* < 0.001.

### Defensive effects of FXE on cardiac histoarchitecture

**Fig. 4:**
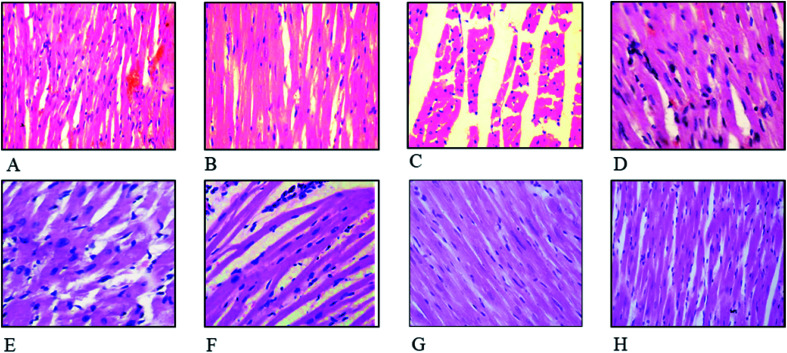
Histopathological studies of the heart. Hematoxylin and eosin stain (40×): (A) control; (B) vehicle treated (DMSO + olive oil) cardiac tissues; (C) CCl_4_ + olive oil; (D) CCl_4_ + silymarin (100 mg kg^−1^); (E) CCl_4_ + FXE (150 mg kg^−1^); (F) CCl_4_ + FXE (300 mg kg^−1^); (G) FXE (150 mg kg^−1^); and (H) FXE (300 mg kg^−1^).

The effect of FXE on cardiac tissues in CCl_4_ administered groups compared to the control are presented in Fig. 4. Thin cardiac tissue sections of the control and vehicle treated group exhibited normal cardiac muscle that possessed a central distinct nucleus and was supplied with blood through the capillaries. Regular continued striations of the myofibrils were observed with a branched appearance, the endocardium and pericardium represented a normal architecture with no signs of inflammatory cells infiltration as shown in Fig. 4A and B. The CCl_4_ treated group indicated damaged heart muscles with distorted capillaries as shown in Fig. 4C. The muscle fibers were degenerated, and sub-endocardial necrosis and edema were seen in the CCl_4_ treated group. In contrast, the FXE low dose (150 mg kg^−1^) to some extant showed protective effects on the cardiac muscles as compared to the CCl_4_ (1 ml kg^−1^) group (Fig. 4D). Although the high dose of FXE (300 mg kg^−1^) and silymarin after CCl_4_ administration exhibited a protective potential close to the control (Fig. 4E and F). FXE alone at both the doses did not alter the normal histology of the cardiac tissues (Fig. 4G and H).

## Discussion

Medicinal plants act as potent sources of pharmaceuticals used in traditional systems all over the world.^36^ In recent years, ethnomedicinal data for plants indigenous to a certain area and traditional formulas made from their different parts have gained the attention of the scientific community throughout the world, as natural alternatives to synthetic drugs. The therapeutic use of plants against various ailments have been utilized for centuries. Plant extracts and their derived compounds play an essential role in drug discovery.^37^ This research details the investigation of the *in vitro* antioxidant potential of *Fraxinus xanthoxyloides* leaves extract/fractions and the extract/fraction with the most significant antioxidant activity was further examined using HPLC and *in vivo* antioxidant profiling.

Polyphenols, especially phenolics and flavonoids, have the potential to scavenge hydroxyl and superoxide free radicals.^38^ Complete antioxidant profiling of *F. xanthoxyloides* leaves was done by performing different *in vitro* antioxidant assays, including scavenging of the DPPH, hydroxyl and nitric oxide radical, the total antioxidant ability, reducing capacity and ferrous ion chelating. FXB demonstrated the best DPPH radical scavenging results followed by FXE and FXM, this result was expected because in our previous study FXB expressed the existence of phenolics as well as flavonoids,^39^ which are well known for their metal chelating ability.^40,41^ FXE (IC_50_ = 7.9 ± 0.9 μg ml^−1^) was also found to be a powerful hydroxyl radical scavenger as this radical can damage different molecules in living cells. FXE also showed the most significant ferrous ion chelating (IC_50_ = 28.2 ± 2.7 μg ml^−1^) and nitric oxide scavenging activity (IC_50_ = 32.5 ± 2.9 μg ml^−1^) among all of the extracts/fractions of *F. xanthoxyloides*, which is a vital mechanism of the phenolic antioxidants. Scientific data on the total antioxidant ability of the *F. xanthoxyloides* leaves by the phosphomolybdenum assay is not available. This assay is quantitative and based upon the number of AAE (μg mg^−1^ of extract). In this investigation FXE showed the highest total antioxidant ability (26.3 ± 2.4 μg AAE per mg sample), which was also observed by Jafri *et al.*,^42^ reporting that the ethyl acetate fraction of the aerial parts of *Hedera nepalensis* possess significant total antioxidant capabilities. Phenolic compounds act as good electron donors and have the potential to reduce the Fe^3+^ ion to the Fe^2+^ ion. In reducing the power assay, FXE and FXB demonstrated the highest reducing potential with 16.2 ± 3.1 and 16.7 ± 2.8 μg GAE per mg sample respectively which is again in agreement with Jafri *et al.*^42^ The substantial antiradical capability of the ethyl acetate fraction might be due to the elevated polyphenols quantities.^39^ This is in agreement with preceding information reported by Sajid *et al.*^43^ who suggested that the medicinal plants possess flavonoids and polyphenols and it is due to these that they show antioxidant abilities, as these antioxidants are well established for their radical scavenging activities. More scientific investigations are required to target the core entity responsible for the great potential in these two different methods.

We established from different antioxidant assays that the potential is possessed by FXE, therefore we decided to proceed with this fraction. Among the phytochemicals, phenolics and flavonoids are acknowledged for their wide range of antioxidant activities. Therefore, polyphenols were selected as the standard for the HPLC profiling of FXE owing to their medicinal properties. Catechin and gallic acid are known for their potential as antioxidants as well as anticancer agents,^44^ caffeic acid has potential as an anticancer agent^45^ and rutin possesses antiviral, antioxidant, antihypertensive and antiplatelet properties.^46^ The phytochemical analysis of FXE was performed by employing reverse phase HPLC and the chromatographic outline was matched with the retention times and absorption spectrum of the reference standards (myricetin, gallic acid, rutin, catechins, kaempferol, quercetin and caffeic acid). The HPLC profile showed that FXE comprises of 54.23 ± 3.41 μg/10 mg of rutin, 7.34 ± 0.93 μg/10 mg of caffeic acid, 8.45 ± 1.12 μg/10 mg of gallic acid and 12.67 ± 2.24 μg/10 mg of catechin (Fig. 1).

It has been well documented that heart tissue is at high risk from reactive radical species which instigates destruction owing to their interactions with cytochrome P_450_ enzymes. As discussed in the previously published literature, in addition to the liver, kidney, and lungs, CCl_4_ is also a very potent cardio-toxin that exerts its pathological consequences by initiating oxidative trauma in cardiac tissues.^47^ It has been observed that CCl_4_ induces fibrosis, cardiac hypertrophy and cardiac cell apoptosis in experimental animals.^48^ CK and CK-MB are biomarkers used to predict adverse cardiovascular occasions such as myocardial infarction and death. These are released by damaged heart tissue.^49^ The present research also entails appraisal of the cardio-protective role of *F. xanthoxyloides* against CCl_4_ mediated cardiac trauma. CCl_4_ induced myocardial injuries, which after oral administration of FXE, not only levelled out the reduced antioxidants, but also protected tissues from lipid peroxidation. This restoring potential was revealed by the normal levels of cardiac biomarkers (CK and CK-MB) and the lipid profile (cholesterol and triglycerides) in the blood sera of rats. Thus, the internal structure of the heart was maintained when there was no lipid peroxidation.

The defensive potential of medicinal plants towards heart injuries can be assessed by estimation of the antioxidant enzyme levels. The levels of CAT, POD, SOD, GST, GSR (Table 3), tissue protein, TBARS, GSH, H_2_O_2_ and DNA fragmentation (Table 4) were disturbed by the CCl_4_ induced toxicity. The levels of endogenous enzymes were restored by the plant and the noxious effects of CCl_4_ were ameliorated by reducing the TBARS level and recovering the normal levels of protein and DNA injuries in the heart tissues. The findings of our study are in accordance with those previously reported by Sahreen *et al.*^50^ who reported the protective role of *Carissa opaca* on the heart of rats in CCl_4_ induced oxidative complications. Comet assay studies of the cardiac cells have shown that CCl_4_ triggered metabolites induce DNA injuries. As a result of this, elongation in the comet length, head and tail length as well as the percentage of DNA in the tail (Table 5) was noted. The increase in these parameters supports the notion that DNA breakages have occurred and there may be destruction in the membranous system of the cardiac cells. These damages account for the cardiac injuries as well as the functional anomalies.

Cardiac histology examination is an imperative parameter used to determine the potential medicinal effects of plants on the cellular components of heart tissue. CCl_4_ causes severe injuries and pathologies to the heart muscles, such as myocardial atrophy, nuclear disintegration in the myocytes and disturbed striations. These anomalies were reversed to their normal state by oral administration of FXE (Fig. 4) which shows that the plant extracts have a strong potential to scavenge radicals, thereby mitigating the oxidative damage. Chang *et al.*^51^ also documented similar cardiac impairments triggered by CCl_4_ while evaluating the healing potential of herbal supplements on the heart. They found that plant extracts were good candidates to inhibit CCl_4_ induced cardiac injuries.

## Conclusions

Our research revealed that the cardio-protective potential of the *F. xanthoxyloides* ethyl acetate fraction (FXE) is probably due to the scavenging of free radicals and the restoration of endogenous antioxidant molecules. This effect appears to be facilitated by natural antioxidants in FXE, which diminish the oxidative damage and lead to normal heart function. Further investigations should be performed to reveal the mechanism responsible for the cardio-protective potential of FXE at the molecular level.

## Author contributions

TY made significant contributions to the experimentation, acquisition and drafting of the manuscript, LJ and AR performed the phytochemistry and *in vitro* works, MS contributed to the experimentation and acquisition of data, and FJ, MM and AR processed data and prepared the manuscript. All authors read and approved the manuscript before submission.

## Availability of data and materials

This manuscript contains all of the data.

## Conflicts of interest

The authors declare that they have no conflicts of interest.

## Supplementary Material

## References

[cit1] Brewer M. S. (2011). Compr. Rev. Food Sci. Food Saf..

[cit2] Shen B. (2015). Cell.

[cit3] El-Baz F. K., Khalil W. K. B., Aly H. F., Shoman T. M., Saad S. A. (2015). Int. J. Pharm. Sci. Rev. Res..

[cit4] Li P.-C., Chiu Y.-W., Lin Y.-M., Day C. H., Hwang G.-Y., Pai P., Tsai F.-J., Tsai C.-H., Kuo Y.-C., Chang H.-C., Liu J.-Y., Huang C.-Y. (2012). Evid.-Based Complementary Altern. Med..

[cit5] Rechnagel R. O., Glende E. A., Dolak J. A., Waller R. L. (1989). J. Pharmacol. Exp. Ther..

[cit6] Farías J. G., Molina V. M., Carrasco R. A., Zepeda A. B., Figueroa E., Letelier P., Castillo R. L. (2017). Nutrients.

[cit7] Liu Z., Ren Z., Zhang J., Chuang C. C., Kandaswamy E., Zhou T., Zuo L. (2018). Front. Physiol..

[cit8] Pepping J. (1999). Am. J. Health-Syst. Pharm..

[cit9] Shaker M. E., Zalata K. R., Mehal W. Z., Shiha G. E., Ibrahim T. M. (2011). Toxicol. Appl. Pharmacol..

[cit10] Rao P. R., Viswanath R. K. (2007). Exp. Clin. Cardiol..

[cit11] Post-White J., Ladas E. J., Kelly K. M. (2007). Integr. Cancer Ther..

[cit12] Deep G., Agarwal R. (2007). Integr. Cancer Ther..

[cit13] Razavi B. M., Karimi G. (2016). Iran. J. Basic Med. Sci..

[cit14] Quattrocchi U. , CRC World Dictionary of Medicinal and Poisonous Plants: Common Names, Scientific Names, Eponyms, Synonyms, and Etymology, Taylor and Francis Group, Boca Raton FL, 2012, vol. 8, p. 275

[cit15] Shah S. M., Hussain F. (2012). J. Med. Plants Res..

[cit16] Sharma P. K., Sethi G., Sharma S., Sharma T. (2006). Indian J. Tradit. Know..

[cit17] Sarfraz I., Rasul A., Jabeen F., Younis T., Zahoor M. K., Arshad M., Ali M. (2017). Evid.-Based Complementary Altern. Med.

[cit18] Brand-Williams W., Cuvelier M. E., Berset C. (1995). LWT--Food Sci. Technol..

[cit19] Ilavarasan R., Mallika M., Venkataraman S. (2005). Afr. J. Tradit., Complementary Altern. Med..

[cit20] Dastmalchi K., Damien Dorman H., Oinonen P. P., Darwis Y., Laakso I., Hiltunen R. (2008). LWT--Food Sci. Technol..

[cit21] Umamaheswari M., Chatterjee T. K. (2008). Afr. J. Tradit., Complementary Altern. Med..

[cit22] Fejes S., Blázovics A., Lugasi A., Lemberkovics É., Petri G., Kéry Á. (2000). J. Ethnopharmacol..

[cit23] Zu Y., Li C., Fu Y., Zhao C. (2006). J. Pharm. Biomed. Anal..

[cit24] Sakr S. A., Lamfon H. A. (2012). Life Sci. J..

[cit25] Shyu M.-H., Kao T.-C., Yen G.-C. (2008). Food Chem. Toxicol..

[cit26] Chance B., Maehly A. (1955). Methods Enzymol..

[cit27] Kakkar P., Das B., Viswanathan P. (1984). Indian J. Biochem. Biophys..

[cit28] Habig W. H., Pabst M. J., Jakoby W. B. (1974). J. Biol. Chem..

[cit29] Khan M. R., Marium A., Shabbir M., Saeed N., Bokhari J. (2012). Afr. J. Pharm. Pharmacol..

[cit30] Jollow D., Mitchell J., Zampaglione N. a., Gillette J. (1974). Pharmacology.

[cit31] Iqbal M., Sharma S., Rezazadeh H., Hasan N., Abdulla M., Athar M. (1996). Redox Rep..

[cit32] Lowry O. H., Rosebrough N. J., Farr A. L., Randall R. J. (1951). J. Biol. Chem..

[cit33] Pick E., Keisari Y. (1981). Cell. Immunol..

[cit34] Dhawan A., Bajpayee M., Parmar D. (2009). Cell Biol. Toxicol..

[cit35] Wu B., Ootani A., Iwakiri R., Sakata Y., Fujise T., Amemori S., Yokoyama F., Tsunada S., Toda S., Fujimoto K. (2006). Exp. Biol. Med..

[cit36] Bhatia H., Sharma Y. P., Manhas R., Kumar K. (2014). J. Ethnopharmacol..

[cit37] Hamayun M., Khan A., Khan M. A. (2003). Ethnobotanical Leaflets.

[cit38] Mitjavila M. T., Moreno J. J. (2012). Biochem. Pharmacol..

[cit39] Younis T., Khan M. R., Shah N. A., Zai J. A., Khan H. (2016). J. Chem. Soc. Pak..

[cit40] Dorman H. D., Koşar M., Kahlos K., Holm Y., Hiltunen R. (2003). J. Agric. Food Chem..

[cit41] Shah N. A., Khan M. R., Naz K., Khan M. A. (2014). BioMed Res. Int..

[cit42] Jafri L., Saleem S., Kondrytuk T. P., Haq I. u., Ullah N., Pezzuto J. M., Mirza B. (2016). Phytother. Res..

[cit43] Sajid M., Khan M. R., Shah N. A., Shah S. A., Ismail H., Younis T., Zahra Z. (2016). BMC Complementary Altern. Med..

[cit44] Zhao B., Hu M. (2013). Oncol. Lett..

[cit45] Shabbir M., Syed D. N., Lall R. K., Khan M. R., Mukhtar H. (2015). PLoS One.

[cit46] Yang J., Guo J., Yuan J. (2008). LWT--Food Sci. Technol..

[cit47] Eshaghi M., Zare S., Banihabib N., Nejati V., Farokhi F., Mikaili P. (2012). Journal of Basic and Applied Scientific Research.

[cit48] Li P.-C., Chiu Y.-W., Lin Y.-M., Day C. H., Hwang G.-Y., Pai P., Tsai F.-J., Tsai C.-H., Kuo Y.-C., Chang H.-C. (2012). Evid.-Based Complementary Altern. Med..

[cit49] Santos E. S. d., Baltar V. T., Pereira M. P., Minuzzo L., Timerman A., Avezum Á. (2011). Arq. Bras. Cardiol..

[cit50] Sahreen S., Khan M. R., Khan R. A., Alkreathy H. M. (2014). BMC Res. Notes.

[cit51] Chang H.-C., Chiu Y.-W., Lin Y.-M., Chen R.-J., Lin J. A., Tsai F.-J., Tsai C.-H., Kuo Y.-C., Liu J.-Y., Huang C.-Y. (2014). Chin. J. Physiol..

